# 5-Aminolevulinic Acid-Induced Protoporphyrin IX Fluorescence Imaging for Tumor Detection: Recent Advances and Challenges

**DOI:** 10.3390/ijms23126478

**Published:** 2022-06-09

**Authors:** Yoshinori Harada, Yasutoshi Murayama, Tetsuro Takamatsu, Eigo Otsuji, Hideo Tanaka

**Affiliations:** 1 Department of Pathology and Cell Regulation, Graduate School of Medical Science, Kyoto Prefectural University of Medicine, 465 Kajii-cho Kamigyo-ku, Kyoto 602-8566, Japan; hideotan@koto.kpu-m.ac.jp; 2 Division of Digestive Surgery, Department of Surgery, Graduate School of Medical Science, Kyoto Prefectural University of Medicine, 465 Kajii-cho Kamigyo-ku, Kyoto 602-8566, Japan; murayama@koto.kpu-m.ac.jp (Y.M.); otsuji@koto.kpu-m.ac.jp (E.O.); 3 Department of Medical Photonics, Kyoto Prefectural University of Medicine, 465 Kajii-cho Kamigyo-ku, Kyoto 602-8566, Japan; ttakam@koto.kpu-m.ac.jp

**Keywords:** 5-aminolevulinic acid, protoporphyrin IX, tumor, photodynamic diagnosis

## Abstract

5-Aminolevulinic acid (5-ALA) is a natural amino acid and a precursor of heme and chlorophyll. Exogenously administered 5-ALA is metabolized into protoporphyrin IX (PpIX). PpIX accumulates in cancer cells because of the low activity of ferrochelatase, an enzyme that metabolizes PpIX to heme. High expression of 5-ALA influx transporters, such as peptide transporters 1/2, in cancer cells also enhances PpIX production. Because PpIX radiates red fluorescence when excited with blue/violet light, 5-ALA has been used for the visualization of various tumors. 5-ALA photodynamic diagnosis (PDD) has been shown to improve the tumor removal rate in high-grade gliomas and non-muscular invasive bladder cancers. However, 5-ALA PDD remains a challenge as a diagnostic method because tissue autofluorescence interferes with PpIX signals in cases where tumors emit only weak signals, and non-tumorous lesions, such as inflammatory sites, tend to emit PpIX fluorescence. Here, we review the current outline of 5-ALA PDD and strategies for improving its diagnostic applicability for tumor detection, focusing on optical techniques and 5-ALA metabolic pathways in both viable and necrotic tumor tissues.

## 1. Introduction

5-Aminolevulinic acid (5-ALA) has been widely used as a tumor-specific photosensitizing agent for photodynamic diagnosis (PDD) of cancers [1]. 5-ALA is a natural amino acid required for heme synthesis. As the enzymatic activity of the heme synthesis pathway is altered in cancerous cells, the heme precursor protoporphyrin IX (PpIX) accumulates in cells upon 5-ALA administration [1,2]. 5-ALA is non-fluorescent; however, 5-ALA-induced PpIX radiates red fluorescence when illuminated with blue/violet light, enabling the use of 5-ALA for specific tumor detection [3,4].

5-ALA PDD for malignant gliomas and non-muscular invasive bladder cancer has been shown to help in improving tumor removal rate [1,5,6]. Furthermore, in feasibility studies, 5-ALA induced PpIX fluorescence has been utilized for the visualization of many other neoplasms such as hepatocellular carcinoma and gastrointestinal cancers [1,2]. However, 5-ALA PDD still presents challenges in terms of enhancing its diagnostic power. The signal intensity of PpIX fluorescence depends on the type and number of tumors; low-grade gliomas (LGGs), invasive margins of high-grade gliomas (HGGs), and gastrointestinal cancers generally emit only weak fluorescence after 5-ALA administration. PpIX fluorescence is reported to be attenuated at the margins of the HGGs, making accurate identification of tumor margins difficult [1,7,8,9,10,11,12]. In tumors that emit relatively weak PpIX signals, tissue autofluorescence derived from collagen and flavin adenine dinucleotide (FAD) affects their ability to be detected [13]. Background autofluorescence also reduces the ability of PpIX to assist in specifically detecting metastatic carcinoma of gastrointestinal cancers in resected lymph nodes (LNs) [14,15]. Furthermore, non-tumorous sites such as inflammatory lesions sometimes radiate non-specific PpIX fluorescence. The purpose of this manuscript is to provide: (1) a brief review of the current 5-ALA PDD strategies for various neoplasms, and (2) an outlook on approaches to enhance the diagnostic power for tumor detection, especially focusing on optical techniques and 5-ALA metabolic pathways in both living and necrotic tumor tissues.

## 2. 5-ALA Metabolic Pathway

### 2.1. Metabolism of 5-ALA in Viable Cancer Tissues

**Figure 1:**
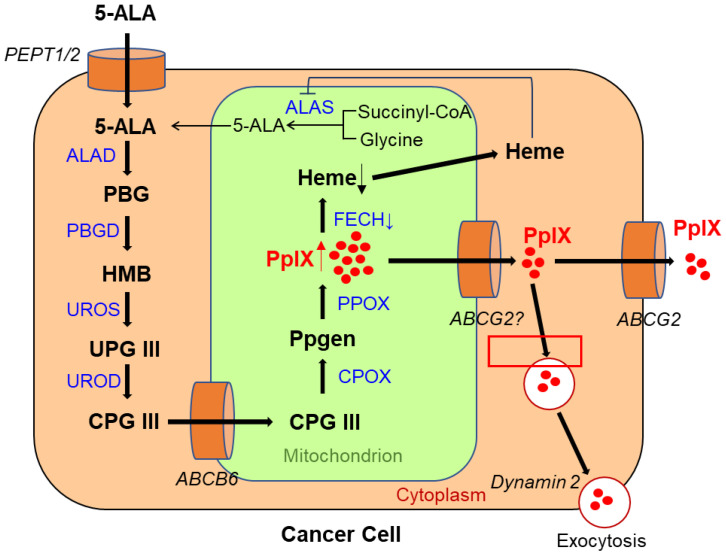
Metabolic pathways of 5-aminolevulinic acid (5-ALA) and mechanism of protoporphyrin IX (PpIX) accumulation in a viable cancer cell. PEPT 1/2, peptide transporter 1/2; *ALAS*, aminolevulinic acid synthase; ALAD, ALA dehydratase; PBG, porphobilinogen; PBGD, porphobilinogen deaminase; HMB, hydroxymethylbilane; UROS, uroporphyrinogen III synthase; UPG III, uroporphyrinogen III; UROD, uroporphyrinogen decarboxylase; CPG III, coproporphyrinogen III; *ABCB6*, ATP-binding cassette transporter B6; CPOX, coproporphyrinogen III oxidase; Ppgen, protoporphyrinogen; PPOX, protoporphyrinogen oxidase; FECH, ferrochelatase; ABCG2, ABC superfamily G member 2.

5-ALA is a natural amino acid and precursor of hemoglobin and chlorophyll [16]. The metabolic pathway of 5-ALA and the mechanism of PpIX accumulation in living cancer cells are shown in Figure 1. Exogenously administered 5-ALA is introduced into the cell via the action of transporters, such as peptide transporters (PEPTs) 1 and 2. Endogenous 5-ALA is produced from glycine and succinyl-CoA in the mitochondria via the enzymatic reaction of 5-ALA synthase (*ALAS)* [17]. Two 5-ALA molecules are condensed by ALA dehydratase (ALAD) to generate porphobilinogen (PBG) in the cytoplasm [17]. Four PBG molecules condense to produce hydroxymethylbilane (HMB) via the porphobilinogen deaminase (PBGD) reaction [17]. In living cancer cells, HMB is converted to uroporphyrinogen III (UPG III) by uroporphyrinogen III synthase (UROS) [17]. UPG III is converted to coproporphyrinogen III (CPG III) through decarboxylation of all acetate groups [17]. CPG III enters mitochondria via ATP-binding cassette transporter B6 (*ABCB6*). In the mitochondria, it is converted to protoporphyrinogen (Ppgen) and then to fluorescent PpIX [17]. PpIX is converted to non-fluorescent heme via the action of ferrochelatase (FECH), which inserts ferrous iron [17]. Moreover, heme negatively regulates *ALAS* activity in some ways, such as by repressing *ALAS* gene expression and degrading *ALAS* mRNA [18,19].

When 5-ALA is administered exogenously to humans, it is rapidly metabolized to heme in normal cells, but in cancer cells, owing to changes in the activity of enzymes in the heme synthesis pathway and/or transporters of 5-ALA and its metabolites, the heme precursor PpIX is selectively accumulated [6,13,20,21]. As anaerobic metabolism in cancer cells is more active than in normal cells, termed the Warburg effect, the tricarboxylic acid cycle is downregulated, resulting in a lack of electrons required to reduce ferric ions to ferrous ions [6]. This leads to the decreased activity of FECH, which transforms PpIX into heme. Therefore, PpIX is selectively accumulated in cancer cells [6,22]. Furthermore, it has been reported that the 5-ALA influx transporter PEPT1/2 is highly expressed in tumor cells, resulting in enhanced PpIX accumulation [23,24,25]. Recently, overexpression of PEPT2 was reported to be related to stronger PpIX fluorescence intensity (FI) in grade 2/3 gliomas; modulation of the function of PEPT2 might thus improve fluorescence-guided resection in grade 2/3 gliomas [25].

**Figure 2:**
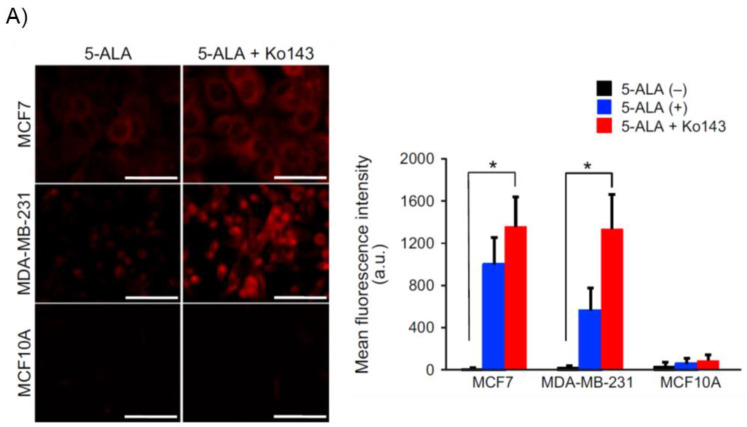

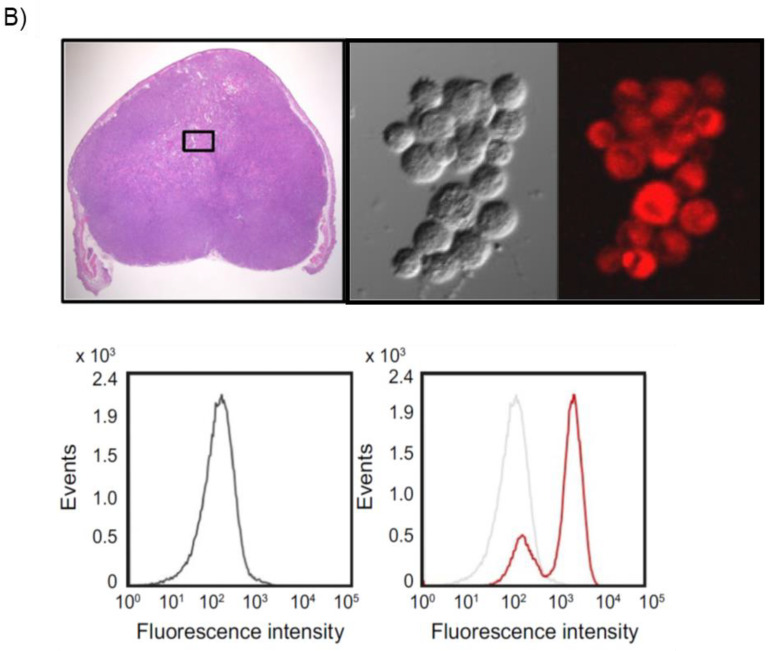
Enhancement of protoporphyrin IX (PpIX)-fluorescence intensity (FI) with an ABC superfamily G member 2 (ABCG2) transporter inhibitor Ko143 in human breast cancer cells and xenografts. “Reprinted/adapted with permission from Ref. [26]. 2019, John Wiley & Sons Ltd”. (**A**) Evaluation of PpIX fluorescence in cultured breast cancer cells (MCF7 and MDA-MB-231) and non-cancer cells (MCF10A) after exposure to 5-aminolevulinic acid (5-ALA; 5 mmol/L for 2 h) with or without Ko143 (1 μmol/L). Confocal fluorescence images of PpIX (left panels). Bars: 50 μm. Comparison of mean FI of PpIX (right panel). All error bars represent the SD (*n* = 3). * *p* < 0.05. (**B**) Fluorescent analysis of fine needle aspiration (FNA) samples obtained from human breast cancer xenografts of MDA-MB-231 cells in nude mice. A hematoxylin and eosin image of a subcutaneous xenograft is shown (upper left panel). Differential interference contrast (upper middle panel) and PpIX fluorescence (upper right panel; excitation: 440 nm, emission: 615–645 nm) images of MDA-MB-231 cells collected through FNA after incubation with 5-ALA (5 mmol/L for 2 h) and Ko143 (1 μmol/L). Flow cytometry analyses of the cells with (lower right panel) and without (lower left panel) incubation with 5-ALA and Ko143 are shown. The black and red lines denote the control nontreatment group and the group treated with 5-ALA and Ko143, respectively. The horizontal axis shows PpIX intensity.

The activity of ABC superfamily G member 2 (ABCG2), which is used to discharge PpIX, also affects the PpIX FI (Figure 1) [24]. Figure 2 shows the effect of Ko143, a specific inhibitor of ABCG2, on PpIX FI in human breast cancer cells and xenografts [26]. The combined exposure of cultured MCF-7 and MDA-MB 231 cancer cells to 5-ALA and Ko143 boosts PpIX-FI compared to that in cultured non-cancer MCF10A cells, suggesting an improvement in the accuracy of 5-ALA PDD (Figure 2A) [26]. Increased FI attributed to the combined administration of 5-ALA and Ko143 was also observed in cancer cells acquired by fine needle aspiration in a murine tumor model (Figure 2B) [26]. In addition, clinically used ABCG2-interacting kinase inhibitors, such as lapatinib, gefitinib, sunitinib, and PD169316, have also been reported to increase 5-ALA induced PpIX-FI in triple-negative breast cancer cell lines [27].

*ABCB6*, another transporter found in the outer mitochondrial membrane, was also reported to affect PpIX FI in malignant cells. Specifically, *ABCB6* is required for the transport of CPG III from the cytoplasm to the mitochondria (Figure 1) [21,28]. Zhao et al. investigated the expression of *ABCB6* in surgical specimens of human gliomas [29]. The results showed that the levels were significantly elevated in human gliomas relative to those in normal brain tissues and that they corresponded to the World Health Organization (WHO) histologic grade [29]. *ABCB6* mRNA expression in distinctly fluorescent tumor tissues was higher than that in faintly fluorescent tumors, suggesting that *ABCB6* might be responsible for PpIX accumulation in glioma cells [29].

Exocytosis, mediated by dynamin 2, a cell membrane-associated molecule, has been reported to influence PpIX accumulation (Figure 1). Kitajima et al. showed a causal relationship between dynamin 2 expression and PpIX excretion in neoplastic cells using a panel of 39 human cancer cell lines from nine different tissues [30]. Moreover, dynamin inhibitors significantly suppress PpIX excretion and increase its intracellular levels [30].

### 2.2. Metabolism of 5-ALA in Tumor Necrotic Tissues

**Figure 3:**
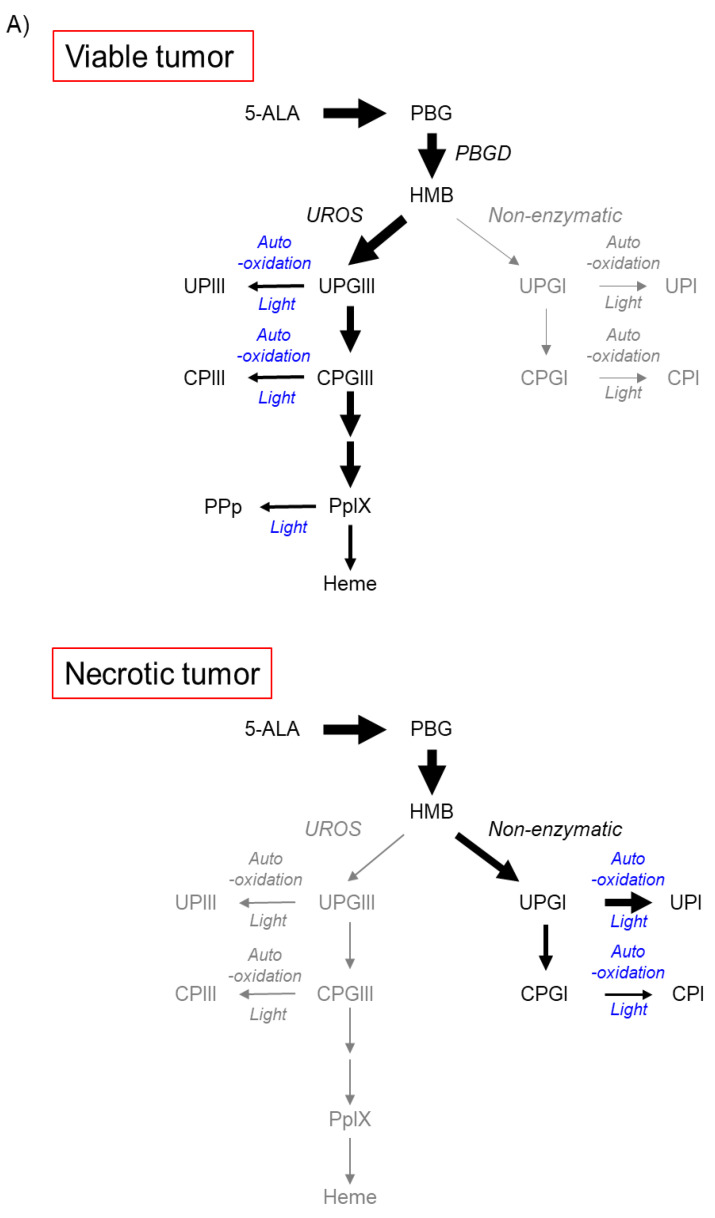

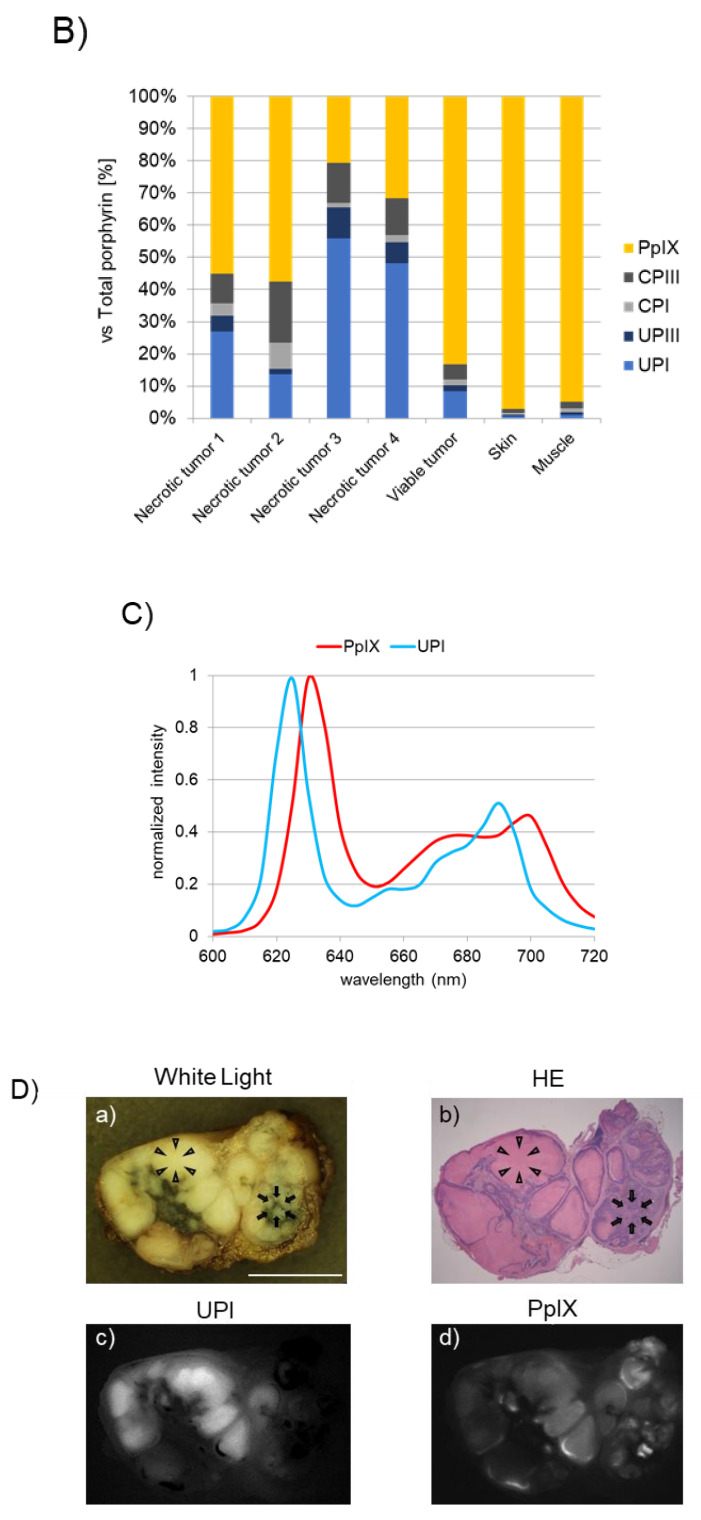
Accumulation of uroporphyrin I (UPI) in necrotic tumor tissues after administration of 5-aminolevulinic acid (5-ALA). “Reprinted/adapted with permission from Ref. [17]. 2021, MDPI”. (**A**) Metabolic pathways of 5-ALA in viable and necrotic tumor tissues. Black and gray arrows represent mainstream and non-mainstream routes, respectively. UPI, UPIII, CPI, and CPIII are produced from corresponding porphyrinogens via autooxidation and/or photocatalysis. PBG, porphobilinogen; PBGD, porphobilinogen deaminase; HMB, hydroxymethylbilane; UROS, uroporphyrinogen III synthase; UPGIII, uroporphyrinogen III; CPGIII, coproporphyrinogen III; PpIX, protoporphyrin IX; UPIII, uroporphyrin III; CPIII, coproporphyrin III; PPp, photo-protoporphyrin; UPGI, uroporphyrinogen I; CPGI, coproporphyrinogen I; CPI, coproporphyrin I. (**B**) High-performance liquid chromatographic measurement of a subcutaneous B88 squamous cancer murine model. 5-ALA hydrochloride (250 mg/kg body weight) was administered intraperitoneally six hours before the experiment. The abundance ratio of each porphyrin to the total dose of porphyrin in necrotic and viable tumors and normal skin and muscle tissues excised from the murine model is depicted. Porphyrins including PpIX, CPIII, CPI, UPIII, and UPI were measured. (**C**) Fluorescence spectra of UPI and PpIX pure chemicals. (**D**) Imaging analyses of an excised metastatic lymph node (LN) consisting of necrotic and viable tumor cells in a patient with esophageal cancer. 5-ALA (15–20 mg/kg body weight) was orally given prior to surgery. White-light image (**a**), hematoxylin–eosin (HE)-stained image (**b**), and spectral unmixed fluorescence images of UPI (**c**) and PpIX (**d**) are shown. The LN was cut in half. Necrotic (arrowheads) and viable tumor tissues (arrows) were observed. Note that tumor necrosis and viable tumor tissue could be visualized with UPI and PpIX images, respectively (**c**,**d**). Scale bar, 5 mm.

The metabolic pathway of 5-ALA in tumor necrosis in metastatic LN disease in patients with advanced esophageal cancer has recently been reported [17]. Tumor necrosis is a feature that often occurs in advanced solid tumors and has been proposed to be a sign of poor prognosis for various types of tumors [31]. In tumor necrosis, 5-ALA administration predominantly results in the production of uroporphyrinogen I (UPGI), but not UPGIII, via a non-enzymatic pathway, probably because of the lack of UROS enzyme activity (Figure 3A) [17].

Figure 3B shows the analytical results of high-performance liquid chromatography in a subcutaneous squamous cell cancer murine model, where the abundance ratio of each porphyrin to the total dose of porphyrin was described [17]. UPI was found in necrotic tumors, whereas PpIX was detected principally in viable tumors, skin, and muscle tissues. UPI was readily produced from UPGI via autooxidation and/or photocatalytic reactions (Figure 3A) [17]. The fluorescence spectra of pure UPI and PpIX showed main peaks at approximately 620 nm and 635 nm, respectively (Figure 3C) [17]. Figure 3D shows the analytical results of metastatic squamous cancer with necrosis in a resected human LN. The locations indicated by arrows and arrowheads show viable and necrotic tumor lesions, respectively (a and b in Figure 3D) [17]. Unmixed fluorescence images of UPI and PpIX enabled the distinction of necrotic lesions from viable lesions (c and d in Figure 3D) [17]. Fluorescence peaks of tumor necrosis and living tumors were observed at 620 nm and 635 nm, respectively (data not shown) [17]. In summary, in viable tumor tissues, voluminous PpIX is produced in viable tumor tissues because the UPGIII/CPGIII axis of the heme synthesis pathway is active (upper panel of Figure 3A) [2,17,32]. In contrast, in tumor necrosis, the spontaneous non-enzymatic route from HMB to UPGI precludes the one from HMB to UPGIII, leading to increased production of UPGI, a precursor of fluorescent UPI (lower graph of Figure 3A) [17,33,34]. In the field of brain surgery, an association between false-positive 5-ALA-induced red fluorescence and radiation necrosis has been reported [35,36]. Although future studies are needed, UPI-derived red fluorescence may be a candidate for the origin of red fluorescence in radiation-associated necrosis.

## 3. 5-ALA-PDD

5-ALA-PDD has been widely used in recent years for enabling fluorescence diagnosis of brain tumors and bladder cancer because tumor tissues specifically emit red fluorescence at approximately 635 nm after 5-ALA administration. However, the efficacy of 5-ALA-PDD in other cancers remains at a preliminary stage.

### 3.1. Gliomas and Other Brain Neoplasms

#### 3.1.1. Gliomas

Complete surgical removal of a glioma is challenging because of its high local invasive potential. Recently, the maximal resection of gliomas has become the standard of care. Progression-free survival and overall survival have improved with greater surgical resection in glioblastoma [37,38]; the most malignant gliomas are called glioblastomas. In 1998, Stummer et al. first reported the usefulness of 5-ALA PDD in the resection of malignant gliomas [39]. In 2006, they reported a randomized controlled multicenter study on fluorescence-guided surgery with 5-ALA for resection of malignant gliomas [40]. The results showed that the use of 5-ALA boosted the complete removal rate (65% versus 36%) and prolonged the 6-month progression-free survival (41.0% versus 21.1%) compared to surgery based on white-light imaging [40]. Furthermore, when total resection using 5-ALA PDD was achieved, the median survival was prolonged by approximately five months [41,42].

5-ALA PDD has mainly been developed for HGGs, which are grade 3/4 tumors according to the WHO classification, and has been approved for use worldwide. In 2007, the European Medicine Agency approved the use of 5-ALA for the detection of malignant tissues during glioma surgery [43,44]. In 2013, 5-ALA was approved as a PDD in Japan. In the USA, the U.S. Food and Drug Administration approved oral 5-ALA in 2017 for use as a perioperative optical imaging agent in patients with gliomas [44]. 5-ALA has a high sensitivity for detecting the main part of HGGs [3,44,45,46,47,48]. However, the specificity has been reported to be low [44,49,50,51,52].

Neoplastic cells in HGGs infiltrate several centimeters away from the tumor mass owing to their invasive characteristics [53]. When biopsies are performed at the invasive margins of HGGs, the detection rate of PpIX fluorescence is low because of the low number of tumor cells [7], which is regarded as a limitation of 5-ALA PDD for HGG detection. Further improvements in measurement methods are required.

In 2020, oral administration of 5-ALA was reported to be useful for the isolation and characterization of circulating tumor-derived extracellular vesicles (EVs) in patients with glioblastoma [54]. EVs are tiny vesicles of cellular origin, containing lipids, proteins, and miRNAs, and can be applied in performing liquid biopsies [54]. 5-ALA-induced PpIX fluorescence seems to have the potential to help identify glioblastoma-derived EVs in blood-based liquid biopsy [54].

Regarding the 5-ALA PDD of LGGs, the present technology is limited by the low PpIX FI within pure LGG [8]. LGGs are grade 1/2 tumors according to the WHO classification. However, anaplastic foci within the LGG have been reported to exhibit PpIX fluorescence [8,55]. Therefore, 5-ALA fluorescence technology appears to be useful for visualizing malignant lesions coexisting with LGG [8,55].

#### 3.1.2. Other Brain Neoplasms

5-ALA-induced PpIX fluorescence is reported to be present in brain metastases and meningiomas [55,56,57,58,59,60,61,62]. However, the usefulness of this method for tumor detection should be considered preliminary compared to that for malignant gliomas. Further studies are needed to evaluate the usefulness of 5-ALA PDD for brain metastasis and meningiomas.

Kamp et al. reported a medical trial of 5-ALA PDD for metastatic brain tumors in 84 patients; 34 cases of metastases (40.5%) showed either strong or faint PpIX fluorescence, and 50 cases of metastases (59.5%) showed no PpIX signal [57]. Subsequently, another clinical study on 5-ALA PDD of brain metastasis consisting of 16 patients showed that 5-ALA-induced fluorescence was more frequently observed in the 14 peritumoral brain tissues (87.5%) than in the five main tumor masses (31.3%), and the existence of the fluorescence in the surrounding brain tissue was related to the deeper invasion of cancer cells [56]. Although it might be partly helpful for the identification of invading metastatic cancer cells, which could make a modest contribution to allow a reduction in the recurrence rate of tumors after surgery, the sensitivity of 5-ALA PDD for brain metastasis does not seem to be high [56].

Residual intracranial meningioma tissues that linger on despite assumed complete resection are often responsible for tumor recurrence. Regarding 5-ALA PDD for meningiomas, Millesi et al. analyzed PpIX fluorescence in 204 surgical cases and observed fluorescence in tumors in 91% of cases [58]. Knipps et al. evaluated tumor invasion of the dura mater around meningiomas using a fluorescence-guided surgical microscope and spectrometer [59]. The sensitivity of the spectrometer was 95% for the visualization of meningioma cells, although observation using a fluorescence-guided surgery microscope failed to visualize residual neoplastic cells in more than half of the cases [59]. Therefore, 5-ALA spectroscopic analysis using the spectrometer seems to be effective for the evaluation of infiltrating cells in the dural tail area owing to its high sensitivity. It is also reported to be useful for the evaluation of bone and brain infiltration in highly malignant meningiomas [60,61].

### 3.2. Bladder Cancer and Other Urological Tumors

#### 3.2.1. Bladder Cancer

Cystoscopy is useful for the diagnosis of bladder cancer; however, some lesions are difficult to visualize using conventional white-light cystoscopy [63,64,65]. For example, flat lesions associated with papillary carcinomas and carcinoma in situ (CIS) can be overlooked. In 1994, Kriegmair et al. reported that PDD following intravesical instillation of 5-ALA was superior to traditional cystoscopy with a white light source in detecting flat lesions, such as tiny papillary tumors and CIS. In 15 patients, 26 neoplastic lesions were diagnosed only by PpIX fluorescence, suggesting that 5-ALA-PDD can support the complete resection of bladder tumors [66,67]. Thereafter, many clinical trials have been conducted and their safety and usefulness have been demonstrated [68,69]. Because 5-ALA is a water-soluble molecule, a fat-soluble ester molecule for intravesical topical administration, hexaminolevulinate hydrochloride (HAL), was developed to further improve PpIX accumulation in tumor cells [6]. In 2003, a multicenter study demonstrated that HAL was effective in detecting superficial bladder cancer, especially CIS [70]. HAL was approved in Europe in 2005 and the United States in 2010 [71]. In Japan, an oral 5-ALA diagnostic agent was approved for the visualization of non-muscular invasive bladder carcinoma during transurethral resection of bladder tumors (TURBT) in 2017 [72]. In 2018, a prospective, multicenter, non-randomized phase III study of oral 5-ALA-PDD for noninvasive bladder cancer demonstrated that its sensitivity (79.6%) was significantly higher than that of conventional cystoscopic diagnosis using a white light source (54.1%) [73]. However, the specificity of the former was lower than that of the latter (80.6% vs. 95.5%) [73]. Taken together, 5-ALA-PDD significantly improves the diagnostic accuracy of non-muscular invasive bladder cancer, especially the CIS detection rate, and improves recurrence-free survival with PDD-TURBT [6,74,75,76,77].

Urine cytology is used for cancer detection in patients with suspected bladder carcinoma and for the post-treatment surveillance of patients with bladder carcinoma. However, its sensitivity is low (55%). The sensitivity is even lower, especially in the case of low-grade bladder cancers [78]. Several preliminary studies on 5-ALA/PpIX fluorescent urine cytology have been reported [79,80,81,82]. Fluorescent urine cytology detects bladder cancer cells by observing PpIX fluorescence after ex vivo incubation of the collected urine specimen with 5-ALA, which is highly sensitive in the diagnosis of bladder cancer and shows a significant difference compared to conventional urine cytology, especially in low-grade bladder cancers [79,80,81]. The development of a new noninvasive 5-ALA/PpIX fluorescent urine cytology system that can be used in routine clinical practice is expected in the future.

#### 3.2.2. Other Urological Tumors

The application of 5-ALA-induced PpIX for the intraoperative visualization of prostate cancer and upper urinary tract tumors has also been reported [83,84,85,86], but 5-ALA PDD of the tumors is still in the preliminary phase, and further studies are needed. Regarding prostate cancer, two pilot studies reported the application of 5-ALA PDD to identify the surgical margin status after radical prostatectomy [83,86]. According to previous studies, the sensitivity and specificity of 5-ALA PDD are 56.0–75.0% and 87.3–91.6%, respectively, and heat degeneration has a crucial influence on PpIX fluorescence [83,86]. The authors concluded that further studies are needed in the application of 5-ALA PDD to radical prostatectomy.

5-ALA PDD has also been reported to be useful for the diagnosis of upper urinary tract tumors, including CIS lesions, in a preliminary prospective single-center trial [6,87]. In an analysis of 31 biopsy specimens, the sensitivity of 5-ALA PDD ureterorenoscopy was apparently higher than that of conventional ureterorenoscopy using a white light source. Specifically, five cases of CIS were visualized only by 5-ALA PDD ureterorenoscopy [6,87].

### 3.3. Digestive Organ Cancer and Other Malignancies

5-ALA-induced PpIX fluorescence has been detected in digestive organ cancers, such as liver and gastric cancers and peritoneal carcinomatosis [12,88,89,90,91,92]. At present, 5-ALA PDD is a promising tool for detecting liver cancers, especially hepatocellular carcinoma. In contrast to tumor imaging, PpIX fluorescence is reported to be very useful for the sensitive detection of bile leakage during hepatectomy because 5-ALA is a bile excretory-type molecule [93].

#### 3.3.1. Liver Cancers

Inoue et al. reported the results of a medical trial of 70 patients who received hepatic resection using 5-ALA-mediated PDD for hepatomas [89]. According to the report, a 5-ALA-induced PpIX signal was found in all hepatocyte cancer cases with serosal invasion; in liver metastasis from bowel cancer cases with serosal invasion, cancer was detected in 18 patients (85.7%) [89]. Tumors remaining at the cut surface after hepatectomy using 5-ALA PDD were less frequently observed than those observed using conventional white-light observation [89]. In addition, all malignant neoplasms were fully resected with a margin with 5-ALA PDD, with an apparent discrepancy in the surgical margin width between 5-ALA PDD (6.7 ± 6.9 mm) and white-light visualization (9.2 ± 7.0 mm; *p* = 0.0083) [89]. Kaibori et al. also demonstrated the usefulness of 5-ALA PDD combined with indocyanine green (ICG) PDD for the intraoperative detection of superficial hepatic tumors that cannot be identified by computed tomography/magnetic resonance imaging before surgery, and concluded that 5-ALA-induced PpIX fluorescence imaging enhanced specificity for the detection of such tumors compared to ICG fluorescence imaging alone [90]. In addition to tumor imaging in the liver, 5-ALA has been reported to be valuable in the identification of bile leakage lesions. Although it is relatively difficult to detect the site of bile leakage using conventional white-light imaging, surgeons can quickly determine the site of bile leakage after hepatectomy using PpIX fluorescence [89,93,94].

#### 3.3.2. Gastric Cancers

The applicability of 5-ALA PDD for gastric cancer is currently under development. However, there are still many challenges to be addressed before the technique can be applied in clinical practice. PpIX fluorescence navigation is reported to result in good visualization and detection of intestinal-type gastric cancers and high-grade adenomas, and the sensitivity of the former was reported to be 93.3% [12,91,95,96]. In contrast, the sensitivity of 5-ALA-PDD for diffuse-type gastric cancers, such as signet-ring cell carcinoma, is low, which hampers its clinical application in gastric cancer diagnosis. However, evaluation of the expression levels of PEPT1 and ABCG2 seems to be useful in predicting the effectiveness of 5-ALA PDD in stomach cancer, since PEPT1 and ABCG2 seem to be key players in controlling intracellular PpIX levels in stomach cancer cells [97]. In addition, the expression of protoporphyrinogen (PPOX) protein, which is involved in PpIX metabolism (Figure 1), was reported to affect FI in early gastric cancer. Specifically, PPOX protein expression was found to be higher in tubular adenocarcinoma with strong PpIX fluorescence than in signet-ring cell carcinoma with weak expression [98]. Elucidation of the precise mechanisms of PpIX accumulation is required to realize 5-ALA PDD in stomach cancer.

Matsumoto et al. developed a prototype of an ex vivo diagnostic imaging instrument for 5-ALA-assistant automated detection of metastasis in excised LNs of patients with stomach carcinoma [13]. Since 5-ALA PDD has been based on the visual judgement of PpIX signals, it can involve inter- and intra-observer variability owing to cognitive differences [13]. Automated detection using an instrument was introduced to address this challenge. To detect metastasis in 323 LNs sliced to a thickness of 2 mm at the center, the areas under the receiver operating characteristic curves were calculated as 0.909–0.921 [13]. Although the diagnostic performance seems to be insufficient owing to false-positive/negative cases at present, the instrument for the automated detection of LN metastasis using 5-ALA has the potential to be a useful tool for intraoperative diagnosis in the future [13].

#### 3.3.3. Peritoneal Carcinomatosis

Peritoneal carcinomatosis is the seeding of carcinoma into the ventral cavity, secondary to abdominal or extra-abdominal malignancy [99]. Since the intra-abdominal cavity has an intricate structure, and metastases are often relatively small, it is difficult to accurately assess the extent of peritoneal carcinomatosis. Therefore, new methods for detecting small seeding sites are required.

Although diagnostic laparoscopy using white light illumination has traditionally been used to detect small foci of peritoneal dissemination, several studies have examined the usefulness of 5-ALA-induced fluorescence. Currently, 5-ALA PDD is in clinical trials, and its usefulness remains undemonstrated. Two clinical pilot studies on staging fluorescence laparoscopy for advanced gastric cancer using 5-ALA have been reported [100,101]; the studies showed that peritoneal metastases and superficial liver micrometastases could be found by 5-ALA PDD. Kishi et al. subsequently reported that diagnostic laparoscopy with 5-ALA PDD improves the sensitivity for the visualization of peritoneal seedings compared to conventional white-light examination [102]. However, they also noted that the detection of peritoneal dissemination in signet-ring cell carcinomas was difficult [101]. Almerie et al. reviewed 12 clinical studies using PDD for the detection of peritoneal carcinomatosis and reported that 5-ALA PDD increases the detected rate of peritoneal dissemination by 21–34% compared to that with white light alone [99]. False positives were found in 0–19% of acquired samples, and most false-positive cases were due to inflammation, granulomatous nodules, or endometriosis [99]. Yonemura et al. demonstrated a detection rate of 17% for appendiceal mucinous neoplasms, 33% for gastric cancer, 54% for colorectal cancer, 67% for diffuse malignant peritoneal mesotheliomas, and 89% for epithelial ovarian cancer in 5-ALA-PDD for peritoneal carcinomatosis [103]. While PpIX fluorescence was detected in all cases of peritoneal dissemination of bile duct cancers, it was not detectable in granulosa cell tumors and gastrointestinal stromal tumors [103]. The authors also reported that PEPT1 and ABCG2 are responsible for specific PpIX fluorescence [103]. Preoperative evaluation of PEPT1 and ABCG2 gene expression may facilitate the selection of patients eligible for 5-ALA-PDD application [104]. Accordingly, the selection of patients based on primary cancer histology and membrane transporter expression might be required for 5-ALA PDD of peritoneal carcinomatosis [103,104].

The development of a new laparoscopic spectrophotometric device to sensitively detect the 5-ALA-mediated PpIX fluorescence spectrum has recently been reported [105]. Although the study was limited to ex vivo analysis, 5-ALA PDD using laparoscopic spectrophotometry has the potential to enhance its diagnostic ability for peritoneal carcinomatosis.

## 4. Challenges in 5-ALA PDD

Although 5-ALA PDD is useful for the detection of HGG and non-muscular invasive bladder cancer, challenges remain for a more precise diagnosis of tumor presence and for the widespread deployment of this method for the intraoperative diagnosis of other tumors. Two topics related to the challenges of 5-ALA PDD are discussed below.

### 4.1. Elimination of Background Autofluorescence

Tissue-derived autofluorescence can hamper PpIX fluorescence detection. For example, the accumulated concentration of PpIX is low in LGGs and marginal lesions of HGGs, making it difficult to analyze PpIX fluorescence because it is masked by background autofluorescence signals derived from FAD, collagen, and other molecules [11]. In addition, digestive tissues exhibit strong collagen-derived autofluorescence, which overlaps with PpIX fluorescence [14,15]. In other words, in tumors in which PpIX fluorescence is relatively weak, it is necessary to distinguish and detect tissue-derived autofluorescence, for which the spectrum overlaps with the PpIX signal. Several analytical methods have been proposed to reduce the effect of background autofluorescence in tumor tissues with weak PpIX fluorescence.

Spectroscopic analysis using a spectrometer is considered simple and useful for objective evaluation and semi-quantification of PpIX FI in tissues accompanied by autofluorescence. The measurement of PpIX fluorescence peak intensity in tumor tissues by fluorescence spectroscopy has been shown to have higher sensitivity and specificity than gross fluorescence evaluation using operating microscopes [106,107,108]; specifically, fluorescence spectroscopy has much higher sensitivity than conventional fluorescence imaging, even allowing for the detection of tumor foci consisting of only 1000 cells [108]. An intraoperative handheld spectrometry system for semi-quantification of PpIX fluorescence has been reported to be useful in clinical practice [109]. However, it is impossible to obtain two-dimensional tumor images using spectrometry alone. Imaging measurements are preferable to point measurements to intuitively grasp detailed information about the entire tumor. Several fluorescence imaging approaches for the specific detection of PpIX fluorescence have been proposed, including spectral unmixing [14,110], the ratio method using photo-protoporphyrin formation [111], the differential method [13], and fluorescence lifetime imaging (FLIM) [4,112,113]. The imaging methods used are described as follows.

#### 4.1.1. Spectral Unmixing

**Figure 4:**
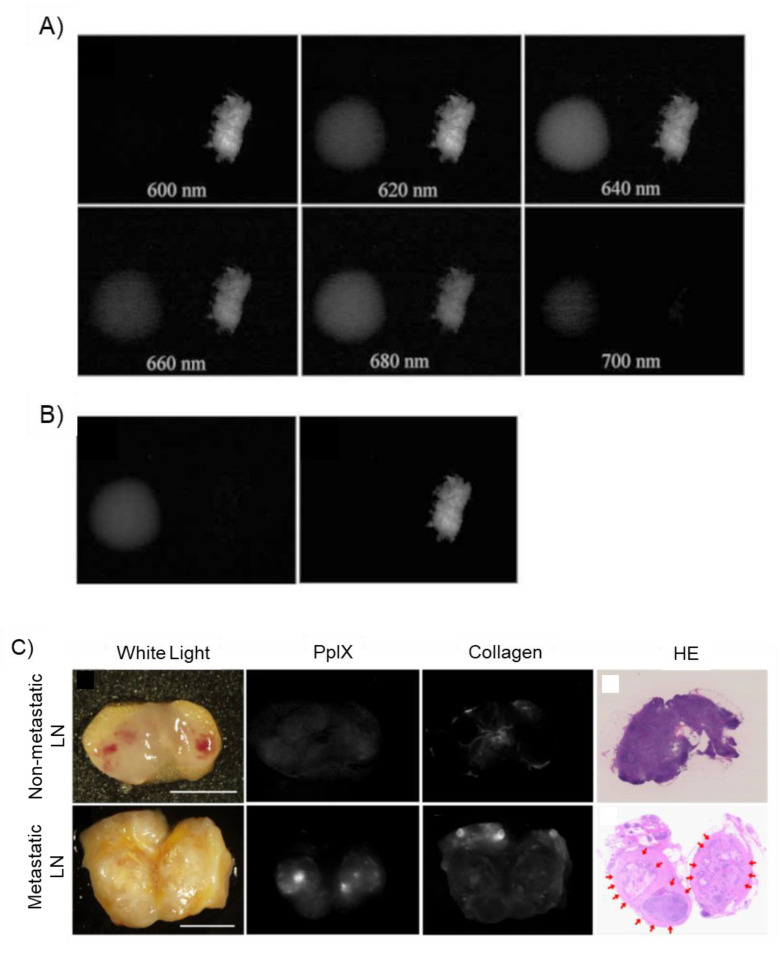
Elimination of background tissue autofluorescence by using a spectral unmixing method for specific visualization of protoporphyrin IX (PpIX) fluorescence. “Reprinted/adapted with permission from Ref. [14]. 2021, MDPI”. Results of imaging of lymph node (LN) metastases in human bowel carcinoma are shown. (**A**) Hyperspectral fluorescence images of pure chemicals of PpIX (left part in each image) and collagen (right part in each image) as references for calculating spectral unmixing. The fluorescence images obtained from 600 to 700 nm at 20-nm intervals are shown. (**B**) Obtained graphics of PpIX (left image) and collagen (right image) after spectral unmixing on the basis of the reference spectral images (**A**). (**C**) Representative images of LNs cut in half: non-metastatic (upper images) and metastatic LNs (lower images) are shown. White-light images (1st row), spectral unmixed fluorescent images of PpIX (2nd row), spectral unmixed fluorescent images of collagen (3rd row), and hematoxylin–eosin-stained images (4th row). Red arrows show metastatic carcinomatous regions. 5-ALA hydrochloride (15 mg/kg of body weight) was administered orally two hours before surgery. Scale bar: 5 mm.

Spectral unmixing is a method to resolve the crosstalk among the fluorescence emission spectra of several materials [14,114]. This methodology presumes that the signal strength of each pixel in an obtained graphic can be shown as a linear combination of fluorescence spectra from multiple substances with known spectra [14,114]. An example of PpIX fluorescence in human colorectal cancer LN metastasis analyzed using the spectral unmixing method is shown (Figure 4) [14]. After fluorescence hyperspectral images of pure chemicals of PpIX and collagen type I from 480 to 700 nm at 20-nm intervals were acquired, the spectral unmixed images (Figure 4B) were computed and created based on the reference spectral images. Fluorescence spectral images of pure PpIX (left in each graphic of Figure 4A) and collagen type I (right in each graphic of Figure 4A) obtained at 600–700 nm at 20-nm intervals are shown for reference [14]. PpIX predominantly radiated fluorescence in the range 620–680 nm and had the strongest signal intensity at 640 nm (Figure 4A) [14]. Collagen also radiates strong fluorescence in the range of 600–680 nm (Figure 4A) [14]. Thus, collagen fluorescence overlaps with PpIX fluorescence when assessed using conventional band-pass filtering [14]. However, the PpIX signal was differentially isolated from collagen one in the computed unmixed images (Figure 4B) [14]. Assuming that the fluorescent component of human colorectal cancer metastatic LNs consists of the sum of PpIX fluorescence and collagen fluorescence, spectral unmixed images were built based on the reference spectral images of pure PpIX and collagen (Figure 4C) [14]. In non-metastatic (upper panels of Figure 4C) and metastatic (lower panels of Figure 4C) LNs cut in half, PpIX unmixed fluorescence was not observed in non-metastatic LNs, but an unmixed PpIX fluorescence signal was observed in metastatic foci [14]. Although the method is relatively time-consuming for obtaining hyperspectral images, the spectral unmixing method is a modified version of the spectroscopic analysis procedure and provides semi-quantitative fluorescence images of PPIX and autofluorescent substances [115].

#### 4.1.2. Differential Method and Ratio Method

**Figure 5:**
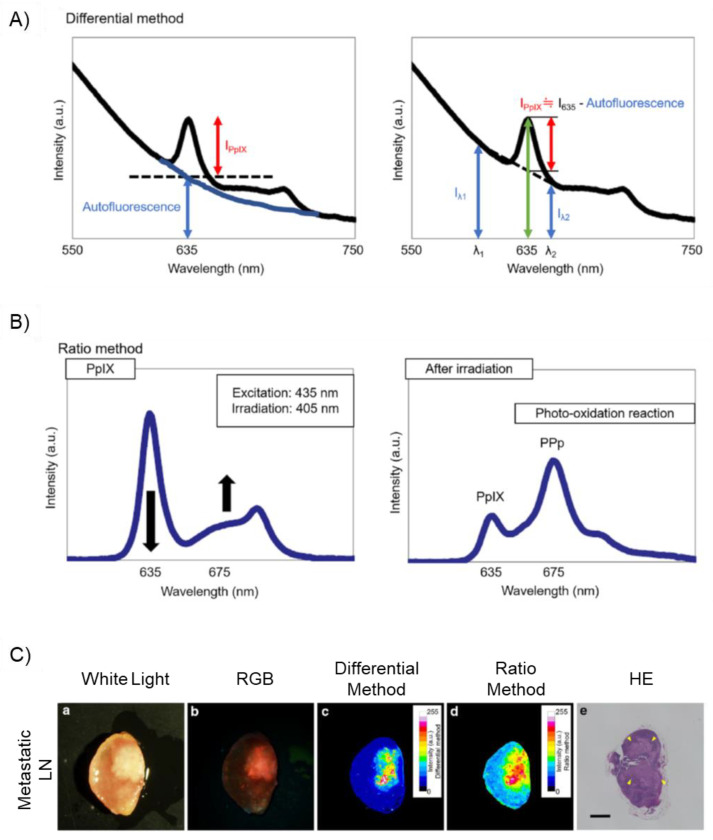
Elimination of background tissue autofluorescence by using the differential method and the ratio method for specific detection of protoporphyrin IX (PpIX) fluorescence. “Reprinted/adapted with permission from Ref. [13]. 2020, Springer”. (**A**) A basic principle of the differential method. The fluorescence intensity (FI) of autofluorescent materials at 635 nm is evaluated by a linear approximation of the spectrum (right panel). PpIX FI at 635 nm is computed by subtracting the FI of autofluorescence materials. (**B**) A basic principle of the ratio method. The photo-protoporphyrin (PPp) fluorescence peak at 675 nm increases and the PpIX fluorescence peak at 635 nm decreases after continuous 405 nm wavelength light irradiation. The increase in the intensity ratio of 675–635 nm before and after light irradiation shows the existence of PpIX. (**C**) Representative images of a metastatic lymph node (LN). 5-ALA (20 mg/kg) was orally administered to patients with stomach cancer prior to surgery. A white-light image (**a**), a fluorescence image obtained with traditional fluorescence microscopy (**b**), calculated images after application of the differential method processing (**c**) and the ratio method processing (**d**), and a hematoxylin–eosin (HE)-stained image (**e**) are shown. The FI value was normalized in both methods. Arrowheads show metastatic lesions. Scale bar: 2 mm.

The differential method is a simple image processing technique that can remove the autofluorescence of background tissues [13,115]. The fluorescence spectrum of tumorous tissue accompanied by a strong background autofluorescence signal after 5-ALA administration is shown in Figure 5A [13]. The FI at 635 nm with blue light excitation is derived from PpIX fluorescence and tissue autofluorescence (left panel of Figure 5A), which are mainly emitted from FAD and collagen [13,115]. To remove unwanted autofluorescence, the fluorescence strengths in the three wavelength ranges were measured, the autofluorescence intensity at 635 nm was evaluated by linear approximation of the spectrum, and the PpIX FI at 635 nm was computed by subtracting the autofluorescence strength (right panel of Figure 5A) [13,115]. This method has the advantages of high-speed imaging owing to the small number of images acquired, protection of the specimen owing to the short total exposure time, and in vivo real-time imaging [115].

To reduce the negative effects of autofluorescence, the photooxidation phenomenon of PpIX can also be used to specifically detect PpIX fluorescence, which is called the ratio method [13,111]. The ratio method depends on the photo-conversion of PpIX to photo-protoporphyrin (PPp) by blue-light irradiation [13]. Upon photoirradiation, PpIX is oxidatively converted to PPp (upper panel of Figure 3A); PPp has a spectral peak at 675 nm and PpIX has a peak at 635 nm [13]. Continuous 405 nm light irradiation of tumor tissue increased the PPp spectral peak at 675 nm and decreased the PpIX spectral peak at 635 nm (left panel of Figure 5B) [13]. Therefore, the intensity ratio of PpIX at 675 and 635 nm can be increased by photoirradiation (right panel of Figure 5B) [13]. The increase in the 675/635 nm intensity ratio after light irradiation shows the presence of PpIX [13]. Although the ratio method is effective in removing the effect of autofluorescence, it requires light irradiation for photo-conversion, and the measurement takes anywhere between several tens of seconds to several minutes.

Figure 5C shows representative images of metastatic LN obtained using differential and ratio methods [13]. In the metastatic LN image obtained using conventional fluorescence microscopy with a red-green-blue charge-coupled device camera, red/orange fluorescence derived from PpIX and autofluorescent substances was observed (second row in Figure 5C) [13]. When the difference and ratio method processing was applied, a specific PpIX signal was observed (third and fourth rows in Figure 5C) [13]. These methods are capable of semi-quantitative analysis of PpIX FI by reducing the influence of autofluorescence [13].

#### 4.1.3. FLIM

**Figure 6:**
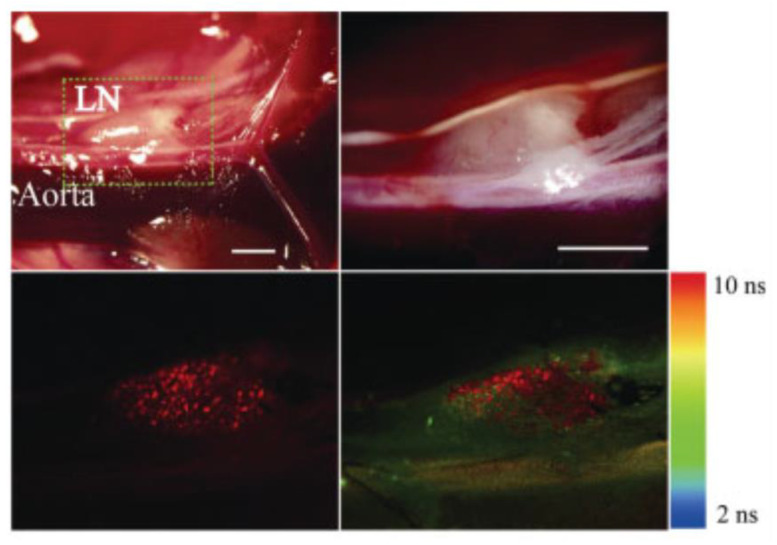
Fluorescence lifetime imaging (FLIM) analyses for specific detection of protoporphyrin IX (PpIX). “Reprinted/adapted with permission from Ref. [4]. 2009, John Wiley & Sons Ltd”. Results of in vivo evaluation of lymph node (LN) metastases in an orthotopic xenograft murine model of human colorectal HT-29 carcinoma cells after treatment with 5-aminolevulinic acid (5-ALA) are shown. 5-ALA (250 mg/kg body weight) was injected in the tail vein before the experiment. A portion of the abdominal cavity, enclosed with a dashed line (upper left: white-light image), was examined using FLIM microscopy. Also depicted are an extended figure of the identical field (upper right: white-light image), a confocal fluorescence intensity image (lower left; excitation: 405 nm, emission: 600–660 nm), and the corresponding FLIM image (lower right; excitation: 405 nm, emission: >420 nm). Scale bars: 1 mm.

One potential tool for specifically detecting PpIX in autofluorescent tissues is time-resolved fluorescence measurement. The fluorescence lifetime is the mean temporal difference between the excitation of fluorescent molecules and their fluorescence emission [113]. In 2009, FLIM analysis of PpIX was performed in a mouse colorectal cancer LN metastasis model after 5-ALA administration [4]; Figure 6 shows the result of in vivo FLIM analysis of LN metastasis in a mouse [4]. The boxed field of a metastatic LN in the peritoneal cavity (upper left graph of Figure 6: white-light image) was observed using FLIM. An enlarged illustration of the boxed field (upper right graph of Figure 6: white light image), confocal FI image (lower left graphic; excitation: 405 nm, emission: 600–660 nm), and the corresponding FLIM image (lower right graphic; excitation: 405 nm, emission: >420 nm) are depicted [4]. The metastatic lesion in the LN, invisible in white-light imaging, can be visualized by sensing the differences between the lifetimes of PpIX and autofluorescent molecules [4]. The fluorescence lifetime of the metastatic carcinoma was found to be longer than that of the background non-metastatic region; the fluorescence lifetime of PpIX was reported to be 16.4 ns, whereas the autofluorescence lifetime of mouse and human tissues is approximately 0.8–2 ns [116,117,118]. Therefore, FLIM analysis can specifically discriminate PpIX molecules from other autofluorescent molecules in the tissues. According to recent reports, PpIX lifetime imaging has the potential to render tumor detection highly sensitive and distinguish tissues emitting weak PpIX fluorescence, such as infiltrated areas of LGGs, from background non-tumor tissues [113,119].

### 4.2. Non-Specific PpIX Accumulation in Non-Tumor Tissues

**Figure 7:**
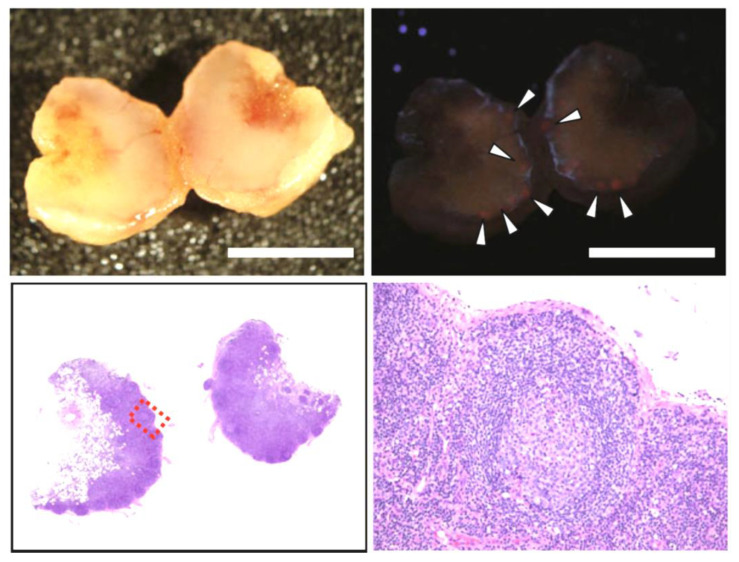
Non-specific deposition of protoporphyrin IX (PpIX) in lymphoid follicles in a non-metastatic lymph node (LN). “Reprinted/adapted with permission from Ref. [15]. 2013, Springer”. 5-ALA hydrochloride (15 mg/kg body weight) was administered orally 2 h before anesthesia induction. A white-light image (upper left panel), a fluorescence image (upper left panel) (excitation: 405 nm; emission: 430 nm), and hematoxylin–eosin (HE)-stained sections (lower panels) are depicted. Arrowheads show red fluorescent nodules originating from accumulated PpIX in lymphoid follicles. The HE-stained images include the lymphoid follicles identical to the fluorescent nodules. The lower right figure is an enlarged drawing of the boxed field shown in the left lower figure. Scale bars: 3 mm.

Another challenge is the non-specific accumulation of PpIX in nontumor tissues. PpIX fluorescence in non-neoplastic tissues has been reported in regions of inflammatory infiltration around HGG, reactive gliosis in patients receiving adjuvant therapy for recurrent HGG, and LNs accompanied by lymph follicles [13,15,55,111,120]; peritumoral inflammatory conditions and elevated reactive mitotic activity could provide an explanation for the false-positive findings [55]. Some researchers have suggested that PpIX deposition in inflammatory lesions is associated with intracellular iron metabolism [107,108]; however, the precise mechanism is unknown. Figure 7 depicts an example of non-specific PpIX accumulation in the lymph follicle of an LN [15]. This LN was obtained from a patient with gastric cancer and cut in half. Although no metastatic carcinoma was observed in the LN, PpIX fluorescence signals were observed in round shapes, consistent with lymph follicles (arrowheads in Figure 7).

Especially in the case of 5-ALA PDD of recurrent HGG, critical interpretation of intraoperative fluorescence is required because non-specific PpIX fluorescence can be seen in inflammatory lesions [55,120]. Additionally, in other neoplasms, it is important to reduce the false-positive rate owing to non-specific PpIX accumulation to improve the performance of 5-ALA PDD. Further studies on the mechanism of non-specific PpIX accumulation in non-tumor tissues are needed.

## 5. Conclusions

Here, we review the current outline and challenges associated with 5-ALA PDD. The 5-ALA PDD for HGG and non-muscular invasive bladder cancer is currently widely in use. However, the diagnosis of other carcinomas remains at the research stage. In tumors with weak PPIX fluorescence, background autofluorescence can interfere with accurate tumor detection, but sophisticated optical approaches can improve the diagnostic power of 5-ALA PDD in clinical practice. In addition, a more detailed analysis of the mechanism of PpIX accumulation in tumorous and non-neoplastic lesions may enhance the efficacy of 5-ALA PDD. We hope that 5-ALA PDD with further technological innovations can be applied in various cancer therapies in the future.

## Data Availability

Not applicable.
